# Recurrent mild cerebral ischemia: enhanced brain injury following acute compared to subacute recurrence in the rat

**DOI:** 10.1186/s12868-016-0263-x

**Published:** 2016-05-26

**Authors:** Ursula I. Tuor, Zonghang Zhao, Philip A. Barber, Min Qiao

**Affiliations:** Department of Clinical Neurosciences and Hotchkiss Brain Institute, Cumming School of Medicine, University of Calgary, Calgary, AB T2N 4N1 Canada; Department of Physiology and Pharmacology, University of Calgary, Calgary, AB T2N 4N1 Canada

**Keywords:** Cerebral ischemia, Transient ischemic attack, Stroke, Recurrence, Inflammation, Granulocytes

## Abstract

**Background:**

In the current study, a transient cerebral ischemia *producing selective cell death* was designated a mild ischemic insult. A comparable insult in humans is a transient ischemic attack (TIA) that is associated with functional recovery but can have imaging evidence of minor ischemic damage including cerebral atrophy. A TIA also predicts a high risk for early recurrence of a stroke or TIA and thus multiple ischemic insults are not uncommon. Not well understood is what the effect of differing recovery times between mild ischemic insults has on their pathophysiology. We investigated whether cumulative brain damage would differ if recurrence of a mild ischemic insult occurred at 1 or 3 days after a first insult.

**Results:**

A transient episode of middle cerebral artery occlusion via microclip was produced to elicit mild ischemic changes—predominantly scattered necrosis. This was followed 1 or 3 days later by a repeat of the same insult. Brain damage assessed histologically 7 days later was substantially greater in the 1 day recurrent group than the 3 days recurrent group, with areas of damage consisting predominantly of regions of incomplete infarction and pannecrosis in the 1 day group but predominantly regions of selective necrosis and smaller areas of incomplete infarction in the 3 days group (P < 0.05). Enhanced injury was reflected by greater number of cells staining for macrophages/microglia with ED1 and greater alterations in GFAP staining of reactive astrocytes in the 1 day than 3 days recurrent groups. The differential susceptibility to injury did not correspond to higher levels of injurious factors present at the time of the second insult such as BBB disruption or increased cytokines (tumor necrosis factor). Microglial activation, with potential for some beneficial effects, appeared greater at 3 days than 1 day. Also blood analysis demonstrated changes that included an acute increase in granulocytes and decrease in platelets at 1 day compared to 3 days post transient ischemia.

**Conclusions:**

Dynamic changes in multiple inflammatory responses likely contribute to the time dependence of the extent of damage produced by recurrent mild ischemic insults. The time of mild stroke recurrence is crucial with early recurrence producing greater damage than subacute recurrence and this supports urgency for determining and implementing optimal stroke management directly after a TIA.

**Electronic supplementary material:**

The online version of this article (doi:10.1186/s12868-016-0263-x) contains supplementary material, which is available to authorized users.

## Background

Ischemic stroke remains a leading cause of death and a major cause of adult disability resulting in a huge personal, social and economic burden [1]. Often considered a warning sign for stroke, a transient ischemic attack (TIA) results from a cerebral ischemic episode of short duration associated with a temporary blockage of a cerebral artery resulting in transient (less than 24 h) functional deficits [2, 3]. Although TIA’s are associated with substantial or full functional recovery, there is both experimental and clinical evidence that reperfused brain may often suffer some permanent damage such as scattered cell death or selective neuronal loss following a transient ischemic event [4–7]. Understanding better the pathophysiology of such mild transient ischemic injury is of some urgency considering an anticipated higher incidence of TIA as the population becomes increasingly elderly, and, an anticipated increasing number of transient cerebral artery occlusions will be successfully recanalized with tissue plasminogen activator or endovascular therapy [8–10].

In addition to being key to treatment approaches, improved knowledge regarding the pathophysiology of a relatively short transient ischemic insult resulting in recovery is also of importance for understanding the interactions of multiple events considering that a TIA is associated with an increased risk of a second or recurrent stroke [2, 3, 11]. A majority of TIA’s are associated with cortical ischemic events and large vessel disease and recurrence is most commonly associated with a second ipsilateral cortical ischemic event [12, 13]. However, surprisingly little is known of the interaction of the ischemic injury processes that occur with multiple mild ischemic insults where the insults are sufficiently severe to cause selective cell death within the ischemic territory. Duration between insults is likely a crucial factor considering that ischemic injury and brain recovery evolve over time. Furthermore, the pathophysiology of early recurrence is important to understand considering recurrence is frequently observed by 1 day after a TIA [14].

Thus the objective of the current study was to determine whether cumulative damage from multiple mild transient ischemic insults, each of sufficient severity to cause selective necrosis, is influenced by the recovery time between insults. A secondary objective was to determine whether the evolution of cellular changes after the first insult were associated with the total damage produced by the recurrent insult. We hypothesized that damage with an early recurrent stroke would be greater than with a subacute recurrent stroke. An animal model was considered key because ischemic severity could be varied to produce mild ischemic cerebral changes as can be observed clinically. Furthermore the timing between insults could be well controlled. Thus we used a rat model of transient focal ischemia [7, 15] adjusted to produce selective cell death, which in young Wistar rats required 30 min of occlusion; this was designated to be a mild transient cerebral ischemic insult. We also produced a recurrence of the insult at 1 or 3 days with the results demonstrating that brain damage is greater when recurrence is acute (1 day) rather than subacute (3 days).

## Methods

Male Wistar rats (Charles River, Montreal, Canada) were acclimatized to a 12 h light/dark cycle with free access to food and water. Animals were randomized to one of three groups involving double surgical procedures: a control group with sham surgery + transient middle cerebral artery occlusion (MCAO) at 1 day and experimental groups with MCAO + recurrent MCAO at 1 day and MCAO + recurrent MCAO at 3 days (n = 6/group). Sample size was selected to minimize numbers of animals used yet provide power as estimated to detect a 30 % difference between the anticipated means of groups assuming a standard deviation of 15 %. Animals were euthanized 7 days after the last surgery and brains were processed for histology to determine the extent of ischemic brain damage. In additional experiments performed to investigate longitudinal cerebral ischemic changes, animals were randomized to MCAO and euthanasia at 1 or 3 days after the transient ischemia (n = 8/group). Peripheral systemic inflammatory changes were also investigated in additional animals from blood sampled at either 1 or 3 days following transient MCAO (n = 5/group).

### Transient mild focal ischemic insult

Surgical procedures were performed aseptically under isoflurane anesthesia and included analgesic measures to alleviate suffering. On the day of the first surgery, a transient mild ischemic insult (mild transient MCAO) was produced in the laboratory by temporarily occluding the distal MCA using a microsurgical approach as described previously [7, 15]. Briefly, a microaneurysm clip was placed on the MCA through a small burr hole in the temporal bone above the MCA where it crosses the rhinal fissure. Rectal temperature was maintained using a servo-controlled heating lamp. A tail artery was cannulated for obtaining arterial blood samples and a small burr hole over the ipsilateral parietal cortex was used to measure cerebral perfusion with laser Doppler flowmetry. Concurrent to the MCAO, both common carotid arteries were transiently occluded using suture thread. At the end of the 30 min occlusion, the microclip and carotid artery ligatures were removed. A dura substitute (Gore Preclude MVP, Better Hospital Supplies Corp., Miami, FL) was positioned on closing to facilitate the production of a subsequent second MCAO. Topical anesthetic in addition to the administration of buprenorphine (0.03 mg/kg, s.c. every 12 h as needed) provided analgesia. During a sham operation, animals underwent the same surgical procedures with the exception of vascular occlusion. Following surgery, rats were housed in separate cages with free access to soft and hard food, water and environmental enrichment. Animals were monitored twice daily for the first 2 days and then daily to ensure good recovery. Animals were subjected to a recurrent mild focal ischemic insult by repeating the 30 min MCAO procedure at either 1 or 3 days of recovery following the initial mild MCAO. The location of the second clip placement on the MCA was immediately distal to the original position to minimize surgical complications.

### Histology to detect brain injury or repair responses

For histological analysis, pentobarbital anesthetized rats were perfusion fixed with formalin and brains were embedded in paraffin. Sections (6 μm) were stained with standard and immunohistochemical methods and altered staining or ischemic injury were assessed blinded to the animal’s identification or surgical group.

### Standard staining with hematoxylin and eosin

In sections stained with hematoxylin and eosin, the extent of cortical damage was identified by classifying ischemic injury in the ischemic middle cerebral artery territory as selective necrosis, incomplete infarction or pannecrosis as described in previous studies [5, 16]. In addition, a score for brain injury was determined [7] by dividing the cortex into 4 regions of interest and a score was assigned to each region graded as: 0 for normal, 1 for <10 % of cellular injury, 2 for 10–50 % of cellular injury, 3 for >50 % cellular injury and, 4 for confluent areas of pannecrosis. A cumulative score was obtained by summing the scores for each region.

### Immunohistochemical staining for cellular ischemic changes

Macrophages/microglia and astrocytes are two glial cell types participating in the CNS inflammatory response to cerebral ischemia. The ischemic changes in these cells observed following 7 days of recovery after multiple ischemic insults also reflect the severity of ischemic injury. Reactive astrocytes were assessed using a primary antibody to glial fibrillary acidic protein (GFAP) (rabbit anti-GFAP, 1:10,000, AB_10013482, Dako, Burlington, ON). Increased activation of macrophages/microglia were assessed using anti-ED1 (AB_2291300, mouse anti-rat CD68 clone ED1, 1:100, AbD Serotec, Raleigh, NC) [17].

The effect of a single mild ischemic insult on the progression of inflammatory changes between 1 and 3 days were assessed first by staining for reactive astrocytes and macrophages/microglia as above. Changes in the cytokine, tumor necrosis factor (TNF) was assessed using goat anti rat TNF-alpha (1:100, AB_354511, R&D system, Minneapolis, MN) [17]. In addition, early activation of microglia was assessed using an antibody against Ionized Calcium-Binding Adapter Molecule 1 (Iba1, 1:1000, AB_839504, Wako Chemicals, USA) which is specifically expressed in microglia and is upregulated following cerebral ischemia [18].

Vascular changes were investigated using a fluorescein isothiocyanate-labeled tomato lectin (1:200, AB_2307440, Vector Laboratories Burlingame, CA) that stains both microglia and vessels [19]. The same sections stained for lectin were also stained using a primary mouse anti-rat endothelial barrier antigen (EBA) IgM (AB_10120605, SMI 71, 1:400, Covance) that stained vessels [20] to assess blood–brain barrier dysfunction. Comparing EBA and lectin stained sections provided an additional detection of morphological and intensity staining changes in microglia. Finally, integrity of the blood–brain barrier was assessed using an antibody to detect extravasation of large plasma proteins (1:200, goat anti rat immunoglobulin G (IgG), Jackson ImmunoResearch Lab, West Grove, PA).

For all the immunohistochemical stains, methods were generally as described previously [7]. Briefly, paraffin sections were first processed for antigen retrieval using citrate buffer at pH = 6, an exception being sections stained with Iba1. The sections were incubated with either 10 % goat or donkey serum, then primary antibody, followed by the appropriate biotin-conjugated IgG (Jackson ImmunoResearch Lab, West Grove, PA) or fluorescent conjugated secondary antibody. For colorimetric staining, horseradish peroxidase conjugated streptavidin (1:400, Dako, Burlington, ON) and diaminobenzidine (Sigma) were applied. For double staining of lectin and EBA, antigen retrieval was omitted and after blocking with goat serum sections were incubated with FITC-lectin at room temperature for 1 h followed by incubation with EBA at room temperature overnight.

### Histological analysis of altered immunostaining

Sections were visualized and analyzed blinded to the experimental group using an Olympus BX61 microscope and Microbrightfield Stereo Investigator (MBF Bioscience, Williston, VT). Immunohistochemical changes were semi-quantified according to the stain used and the types of cellular changes observed in the MCA territory of the parietal cortex ipsilateral and contralateral to the transient occlusion. For ED1 and TNF, regions of parietal cortex within the MCA territory were inspected and cells within at least three fields of view (0.263 mm^2^ each) with positive staining for ED1 or TNF were counted and used to provide a mean value. For EBA, fluorescent images of parietal cortex were captured digitally (200× magnification) and those with technically good vessel staining in the contralateral hemisphere were analyzed using Image J for counting of the number of EBA stained vessels in representative ipsilateral and contralateral fields of view. GFAP stained sections were assigned cumulative scores similar to the hematoxylin and eosin stained sections, with altered levels of staining being scored in each of the four different cortical areas as: 0 for normal, 1 for scattered reactive cells labelled or <10 % of area, 2 for 10–50 % of the area labelled, 3 for >50 % of the area labelled and 4 for confluent areas of pannecrosis with total loss of GFAP stain. A lectin staining score was attained by assessing the extent of increased tomato lectin staining of activated microglia and their processes within the cortical parenchyma (i.e. in excess of vessels labelled with EBA) and scored according to: 0—none, 1—sparse numbers and 2—substantial numbers of parenchymal cells with positive lectin staining. In regions of infarct a score of 3 was assigned if there were substantial numbers of positive stained parenchymal cells in addition to a diffuse increase in signal from within the parenchyma. For Iba1 stained sections, those with the best positive staining of processes were selected for counting both the total number of microglial cells stained per field and the number of activated microglial cells with ramified or thickened bushy processes. Potential increased colorimetric staining in cortex for plasma proteins with IgG were assessed by measuring gray levels in ipsilateral and contralateral cortex along with levels on the blank slide adjacent to the region of interest in order to analyze left–right differences.

### Systemic circulatory response

Hematology analysis of blood samples was also performed in additional rats to assess for signs of immunosuppression or infection. Venous blood samples (0.2 ml) were collected in EDTA coated tubes and analyzed immediately for concentrations of platelets and red and white blood cells (HemaTrue Hematology Analyzer, Heska Corp., Loveland, CO, USA). Relative proportions of the different types of white cells were also assessed by measuring the numbers of monocytes, granulocytes and lymphocytes.

### Statistical analysis

Data were analyzed using SigmaPlot 13 software (Systat Software Inc, San Jose, CA). Data are reported as mean ± SD for groups with continuous values or as the median and first and third quartiles for nonparametric data. Statistical comparisons were considered significant at P < 0.05. Prior to a comparison of means, samples were first tested for normality and equal variance followed by selection of an appropriate analysis of variance and post hoc test (e.g. Bonferroni). Differences between ipsilateral and contralateral hemispheres were compared using a paired Student’s t-test or a Rank Sum Test for categorical values.

## Results

### Brain damage varies with timing between multiple mild insults

**Fig. 1:**
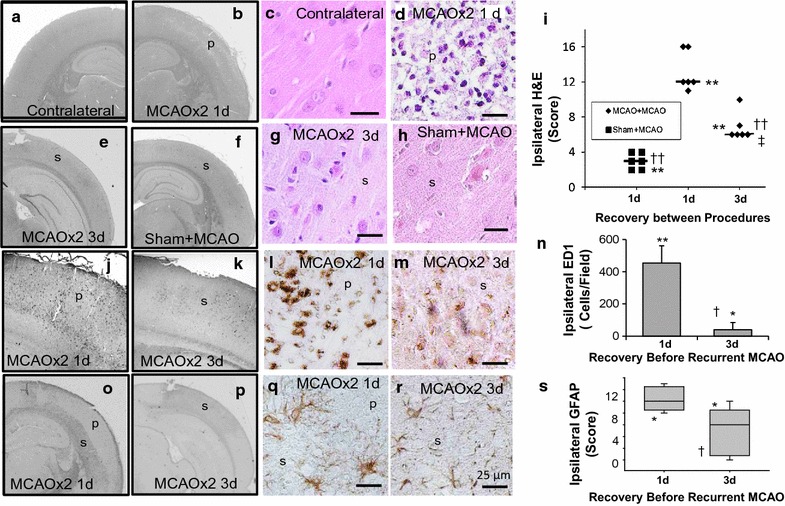
Cerebral injury dependence on recovery time between recurrence of mild transient ischemic insults. Representative cerebral cortical micrographs of sections at low and high magnification (*scale bar* of 25 µm) stained with: hematoxylin and eosin (**a**–**h**), ED1 for activated macrophages/microglia (**j**–**m**), and GFAP for reactive astrocytes (**o**–**r**). Brain was perfusion fixed and processed 7 days following the last insult. Cerebral cortex contralateral to the transient middle cerebral artery occlusion (MCAO) is normal (A,C). Within cerebral cortex (**b**, **d**) after a mild transient MCAO followed by a recurrent MCAO at 1 day there is extensive pannecrosis (*p*). Selective necrosis (*s*) is common following a mild transient MCAO followed by a recurrent MCAO at 3 days (**e**, **g**). Selective necrosis also occurs in rats with a sham surgery followed 1 day later by transient MCAO (**f**, **h**). Semiquantitative assessment of injury scores indicated greater damage with 1 day compared to 3 days recurrence in the H&E sections (**i**). Immunohistochemical staining with ED1 antibody demonstrated an increase in activated macrophages/microglia following recurrent MCAO produced at either 1 or 3 days after an initial MCAO (**j**–**m**). The mean number of cells per field with ED1 staining demonstrating a difference between 1 and 3 days recurrent MCAO groups (**n**). GFAP staining of astrocytes was increased with recurrent MCAO at 1 or 3 days (**o**–**r**). The median score for altered GFAP staining reflected greater injury and astrocytic changes in brains of animals with recurrent MCAO at 1 day than 3 days (**s**). n = 6/group. *P < 0.05; **P < 0.006, Ipsilateral different from contralateral; ^†^P < 0.05; ^††^P < 0.006, different from 1 day Recurrent MCAO. ^‡^P < 0.01, different from Sham

For animals receiving multiple surgical interventions, eight animals were lost to analysis due to mortality post recurrent stroke (n = 1), inadequate flow reduction during clip placement (n = 2), surgical complications (n = 2) and a problem with tissue embedding (n = 2). A total of 18 animals (n = 6/group) were analyzed in the sham + mild MCAO, 1 day recurrent MCAO and 3 days recurrent MCAO groups. Body weights in these 3 groups were similar at baseline and time of the second surgery (290 ± 65 and 282 ± 50; 319 ± 56 and 314 ± 65; 296 ± 45 and 296 ± 40, respectively). In all animals, ischemic changes were observed in the cerebral cortex ipsilateral to the transient middle cerebral artery occlusion (Fig. 1b, d, e–h) but not in the contralateral cortex (Fig. 1a, c). Severity of injury varied between groups (see also data in Additional file 1). Substantial areas of incomplete infarct and pannecrosis in addition to some areas of selective necrosis were apparent within the middle cerebral artery territory in all 6 animals subjected to a recurrent mild ischemic insult separated by 1 day (e.g. Fig. 1b, d). Substantial changes in ED1 and GFAP staining also occurred with recurrence at 1 day post first insult (Fig. 1j, l and o, q, respectively). In contrast, in animals subjected to a recurrent mild ischemic insult separated by 3 days there were only small regions of incomplete infarction with more extensive regions of selective necrosis in four of the six animals and only regions of selective necrosis in 2 animals (e.g. Fig. 1e, g). Positive staining of macrophages/microglia with ED1 or for reactive astrocytes with GFAP was also modest (Fig. 1k, m, p, r). Brains of animals that underwent a sham procedure followed by transient ischemia 1 day later had the least extensive ischemic changes consisting of only selective necrosis (6/6 animals) (e.g. Fig. 1f, h) and minimal staining for ED1 or GFAP (3/3) (Additional file 1).

Semi-quantitative analysis of the stained sections supported these observations (Fig. 1i). All groups had significantly greater damage (median cumulative score ≥3) in the ischemic cortex than in the contralateral hemisphere which was normal (median score of 0). Animals with an early recurrent stroke 1 day after the first insult had significantly greater damage assessed in hematoxylin and eosin stained sections than a sham procedure followed by a mild MCAO. Total damage with early (1 day) recurrence also exceeded that produced by recurrent stroke separated by 3 days (P < 0.05). The differences in severity of damage and their dependence on the recovery time between the first and recurrent insult were also reflected in an increased number of cells staining with ED1 (Fig. 1n) and the severity score for differences in staining for reactive astrocytes with GFAP (Fig. 1s).

### Similar physiological measures in recurrent stroke groups

**Fig. 2:**
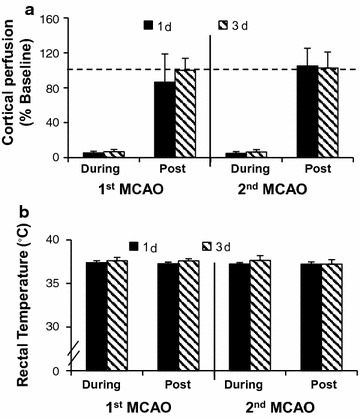
Mean cortical perfusion or rectal temperature during or post middle cerebral artery occlusion (MCAO). **a** Cortical perfusion measured using Doppler flowmetry presented as a percent of baseline. Mean perfusion decreased to <8 % baseline during MCAO subsequently returning toward baseline within the first 5 min following reperfusion irrespective of MCAO recurrence at either 1 or 3 days post the first MCAO (n = 6/group). **b** Mean core body temperature was well controlled resulting in similar means for the recurrent groups either during or after the first or second MCAO

Potential differences in severity of ischemia or other measured physiological parameters that are well known to affect ischemic injury were comparable between groups. Cortical perfusion was less than 10 % baseline during MCAO and reperfusion levels were similar during both the first and second ischemic insult irrespective of the recovery time between them (Fig. 2a). There was also excellent control of body temperature during MCAO and during early reperfusion, irrespective of the experimental group (Fig. 2b). Other physiological variables and blood gases were similar between 1 and 3 days recurrent groups. Mean values were 86 ± 11 and 88 ± 11 mm Hg for mean arterial blood pressure, 102 ± 7 and 109 ± 5 for PO2, 44.2 ± 2 and 38.6 ± 2 mm Hg for PCO2, 7.35 ± 0.01 and 7.36 ± 0.04 for PH and 13.6 ± 3 and 11.6 ± 2 mmol/L for blood glucose, respectively. See also data in Additional file 2.

### Inflammatory changes following a single mild transient ischemia

**Fig. 3:**
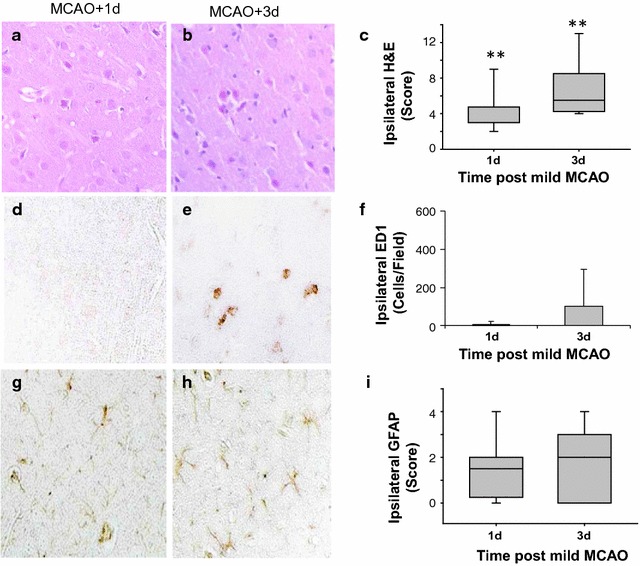
Acute and subacute cortical responses to a single mild ischemic insult. **a**, **b** Hematoxylin and eosin (H&E) stained sections from brains perfusion fixed at 1 or 3 days following a mild transient middle cerebral artery occlusion (MCAO). **c** The median and first and third quartiles of the damage score was not different statistically between groups. **d**, **e** ED1 stained sections from brains 1 and 3 days post MCAO. **f** Mean numbers of ED1 cells stained per field were not different statistically. **g**, **h** GFAP stained sections from animals 1 and 3 days post MCAO. **i** Median and first and third quartiles of the GFAP score were similar between groups (P > 0.05). n = 8/group *P < 0.05; **P < 0.005, ipsilateral different from contralateral. (Mann–Whitney Rank Sum test)

In order to investigate whether differential responses to brain injury and recovery from ischemia could explain the ensuing damage produced by a recurrent MCAO, longitudinal tissue changes to a single mild MCAO were investigated in additional randomized animals. Several of these were lost to analysis due to mortality (n = 1), surgical complications (n = 2) and a problem with tissue embedding (n = 1). Sections from all animals analyzed following a single transient MCAO demonstrated mild damage in hematoxylin and eosin stained sections (Fig. 3a, b). Damage score was similar in the 1 and 3 days recurrent MCAO groups (n = 8/group) (Fig. 3c). In adjacent stained sections, there were increased numbers of ED1 stained macrophages/microglia ipsilaterally in both groups (Fig. 3d, e). There was also altered GFAP staining of reactive astrocytes (Fig. 3g, h) compared to contralateral cortex. These inflammatory changes scored in the 1 and 3 days recurrent groups were not different statistically (Fig. 3f, i). See also data in Additional file 3.

**Fig. 4:**
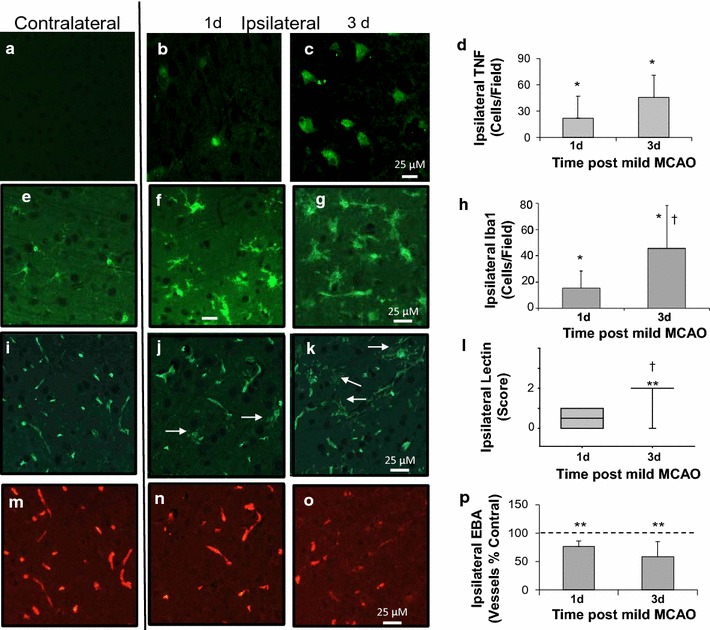
Cerebral Inflammatory and blood–brain barrier marker changes following a single mild transient MCAO. **a** Representative sections stained from contralateral cortex for the cytokine, tumor necrosis factor (TNF). **b**, **c** TNF immunostained sections from ipsilateral cortex at 1 or 3  days post MCAO, respectively. **d** Mean number of TNF stained cells per field is similarly increased at 1 or 3 days post MCAO (n = 8/group). Representative positive staining of microglia with Iba1 in contralateral cortex **e** and ipsilateral cortex at 1 and 3 days post MCAO (**f**, **g**). Activated microglia were observed at 1 day and these were more numerous at 3 days post MCAO (**h**, n = 8/group). Double staining of vessels and microglia with tomato lectin (**i**–**l**) and vessels with endothelial barrier antigen (EBA) (**m**–**p**). Staining of microglia with lectin was minimal in the contralateral cortex (e.g. **i**) and increased (e.g. *arrows*) in ipsilateral cortex at 1 day (**j**) or 3 days (**k**) post transient MCAO. **l** Median scores for lectin staining (n = 8/group). **m** Representative staining of vessels for endothelial barrier antigen (EBA) in contralateral cortex (12 animals with good contralateral positive staining quantified). **n**, **o** EBA staining in ipsilateral cortex from animals at 1 and 3 days post MCAO. **p** Reduced percentage of vessels stained with EBA ipsilaterally relative to contralaterally (n = 6/group). *P < 0.05; **P < 0.005, ipsilateral different from contralateral. ^†^P < 0.01 lectin stained microglia differ between 1 and 3 days post MCAO groups

In sections from these animals we also investigated whether TNF-alpha, often considered to be a pro-inflammatory cytokine, is substantially increased 1 day post a mild ischemic insult and thereby a potential contributor to increased ischemic damage observed with multiple insults at this time. Relative to the contralateral cortex, which had minimal TNF staining (Fig. 4a), increased numbers of positive TNF stained cells were observed ipsilaterally at 1 and 3 days post-insult (e.g. Fig. 4b, c). The mean increased number of TNF stained cells were not different statistically at 1 and 3 days post insult (Fig. 4d). See also data in Additional file 4.

Microglial changes, which are usually considered proinflammatory acutely but can also contribute later to repair [21], were altered following a single mild insult. Microglial cells stained with Iba1 (Fig. 4e–h) were similar in the 1 and 3 days groups contralaterally (e.g. mean of 23.0 ± 6.0 cells per field). Total numbers of cells ipsilaterally increased to 32.4 ± 9 and 64.9 ± 25 in the 1 and 3 days groups respectively (P < 0.05) and included cells with ramified or bushy morphology or round cells (Fig. 4f, g). Those with ramified or bushy morphology were also different between groups (Fig. 4h). See also data in Additional file 4. Using a tomato lectin stain of vessels and microglia, examination of staining in the non-vascular parenchyma contralaterally indicated a lack of appreciable staining for microglial cells with lectin [22] (e.g. Fig. 4i). At 1 day post MCAO there was a modest increase in lectin stained microglia and their processes, associated with microglial activation (e.g. Fig. 4j). At 3 days post-insult, substantial numbers of microglia stained for lectin (e.g. Fig. 4k) and the median scores for lectin staining differed significantly between groups (P < 0.05) (Fig. 4l).

### BBB injury following a mild transient ischemia

In sections stained with lectin, we also performed immunostaining for endothelial barrier antigen (EBA) whose loss is considered to provide a highly sensitive indicator of dysfunction of the blood–brain barrier [20]. There was reduced vascular EBA immunoreactivity observed following a mild ischemic insult. Compared to the contralateral hemisphere (Fig. 4m), there were fewer EBA stained vessels (Fig. 4n–o) and analysis of sections determined a reduced number of EBA stained vessels both at 1 and 3 days post a mild MCAO (Fig. 4p). There was no significant difference between groups. Despite a reduction in EBA staining there was no evidence of major BBB damage. Following a single mild ischemic insult, there was a lack of appreciable positive staining for IgG within parietal cortex (not shown), indicating that the blood–brain barrier was intact to passage of large plasma proteins detectable with IgG. Quantitative analysis of these sections demonstrated there was no difference in the darkness of staining in ipsilateral versus contralateral cortex for the 1 and 3 days groups (−0.7 ± 1.2 and −0.1 ± 1.9 gray levels, respectively) (Additional file 4).

### Systemic inflammation following a mild transient ischemia

**Fig. 5:**
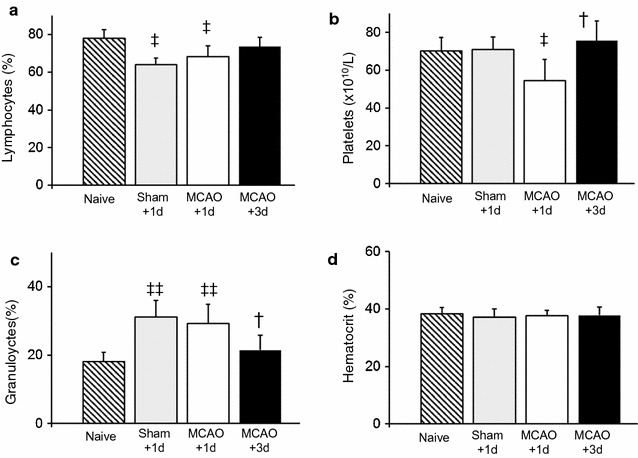
Systemic changes following a Single Mild Transient MCAO. A complete blood count of blood samples from naïve animals or animals at 1 or 3 days post a mild transient MCAO. **a** The concentration of lymphocytes, presented as a % of total white blood cells, was decreased at 1 day post transient MCAO when compared to naïve animals. **b** The mean concentration of platelets was also decreased at 1 day post MCAO. **c** The mean number of granulocytes, normalized to the total number of white blood cells, was transiently increased at 1 day. **d** Mean hematocrit was similar in all groups. N = 5/group. ^‡^P < 0.05; ^‡‡^P < 0.005, different from Naive. ^†^P < 0.05, different from 1 day post MCAO (ANOVA and Bonferroni t-test)

Considering the evidence that systemic inflammation or suppression of the immune system can play a role in influencing ischemic damage [21, 23, 24], we also investigated the potential contribution of systemic immune cell changes to the enhancement of ischemic damage by performing a hematology analysis of blood samples collected at 1 or 3 days following a mild MCAO or a sham surgery. Blood samples from a naïve group of rats provided an additional control (n = 5/group). Groups had a similar hematocrit (Fig. 5d and Additional file 5). Following a mild MCAO there was a decrease in the lymphocyte percentage of white blood cells and the platelet concentration at 1 day but not 3 days relative to naïve controls (Fig. 5a, b). In contrast, there was a transient increase in the granulocyte percentage of white blood cells at 1 day compared to levels either in naïve animals or in blood samples collected 3 days after MCAO (Fig. 5c). Altered lymphocyte and granulocyte concentrations but no change in platelets were also observed 1 day following the stress of a sham surgery. However, the increase in granulocytes alone was insufficient to enhance damage; sham surgery with increased granulocytes and a mild ischemic insult 1 day later produced only mild ischemic changes (Fig. 1c).

## Discussion

The current results demonstrate the major importance of early recurrence of a *mild* transient ischemic insult following an initial TIA. Distinctive in our study, compared to studies of preconditioning, is the investigation of multiple transient ischemic insults—both of sufficient severity to cause mild ischemic damage. We found that a recurrent mild insult following a TIA in rats produced substantially more brain damage when recurrence is acute (i.e. 1 day) than with a subacute recovery time of 3 days between insults. The cause of this difference is likely multifactorial and potentially includes an augmentation of damage by systemic inflammatory changes. Irrespective of the mechanisms, the results demonstrate that the time of mild stroke recurrence can be crucial in influencing brain damage and supports urgency for determining and implementing optimal stroke prevention management early after TIA to avoid a second ischemic event.

### Interaction between two Mild Ischemic Insults

The principal finding of the current study was that the combined damage produced by multiple mild insults was influenced by their timing. In contrast to that in the current study, previous studies of multiple ischemic insults have generally focussed on studies where the first or second insult is a very short preconditioning or postconditioning ischemia that alone produces no permanent cellular damage; such a non-damaging ischemia has protective effects [25–28]. Indeed in the current study the group with a sham procedure prior to transient MCAO had reduced damage compared to that of a single MCAO assessed 3 days later. Using an initial insult that is more injurious, one laboratory has examined the effects of repeated ischemia in the gerbil using only animals that displayed neurological signs of stroke after a first temporary carotid occlusion and subjecting them to additional episodes of the same duration of ischemia [29, 30]. Although small numbers (3–5) per group were investigated, their results suggested a transition from mild to more severe injury with increasing numbers of insults and greater damage when second insults were at 3 or 5 versus 48 h apart. Recently, we used a model of two relatively short transient ischemic episodes, produced by microclip occlusion of the MCA and separated by 3 days to demonstrate an increased damage with recurrent stroke compared to a single insult [15]. The present study also observed an increase in the damage produced by a recurrent compared to a single MCAO, but differences were most marked for an acute (1 day) rather than a subacute (3 days) recurrence. Extensive infarction rather than partial infarction and/or selective necrosis was produced when stroke recurrence was acute rather than subacute. In addition to this finding, we observed several differential systemic and cerebral inflammatory changes at acute and subacute times following a mild transient ischemic insult providing novel insights into the pathophysiology of TIA and potential contributions to recurrent stroke damage. These results are of potential relevance clinically considering an anticipated rise in the incidence of TIA, both due to our ageing population and an escalation in number of ischemic stroke patients that will be treated with early reperfusion therapy [8–10]. Understanding better the pathophysiology of early recurrence is also essential considering that the median time for recurrence of functional deterioration related to a vascular event following a TIA is 1 day [14]. Furthermore, although debilitating strokes are relatively infrequent following a TIA alone, when there is a recurrent event, 53 % have been found to be debilitating [31].

### Physiological and vascular changes

Our physiological measures determined that various factors known to influence infarct size such as body temperature, blood pressure and blood gases were similar between the groups and thus are unlikely to account for the effect of different timing of recurrent insults on brain damage. Although preconditioning ischemia has been observed by others to improve blood flow during ischemia compared to a single severe ischemic insult [32, 33], the levels of cerebral blood flow reduction were equivalent during both insults and for both groups. With respect to reperfusion, there were no significant statistical differences in perfusion within the first 5 min of reperfusion. However, there was a variable return of flow to baseline, with some animals demonstrating hyperperfusion and this variance was associated with suboptimal statistical power requiring caution for deciding the group means were the same. Also a limitation presently was that only *acute* reperfusion was monitored and the possibility remains that at a delayed hypoperfusion could have adversely influenced damage.

Regarding vascular changes, there was little evidence for differences in disruption of the blood brain barrier at early versus subacute times. There was a lack of detectable extravasation of IgG at either time point in agreement with previous reports of a lack of vasogenic edema detectable in T_2_ magnetic resonance images following a mild transient ischemic insult [5, 7]. The reduced staining for EBA did suggest some endothelial injury at both 1 and 3 days post a single MCAO. Early ischemic endothelial injury is associated with an acute activation of the endothelium resulting in an increased expression of endothelial adhesion molecules associated with endothelial injury [21, 24, 34]. However, since reductions in EBA were similar at both time points, there was no direct evidence for greater dysfunction in the blood–brain barrier at 1 than 3 days to help account for greater enhanced damage of a recurrent 1 day insult.

### Cerebral inflammatory changes

We also investigated whether the first mild ischemic insult could produce factors in the tissue that could either augment ischemic damage acutely (i.e. at 1 day) or provide some later (3 days) neuroprotection. The literature is sparse regarding *acute* glial inflammatory changes in the brain following a *mild* ischemic insult mimicking a TIA. At relatively chronic times (1–4 weeks) following a mild focal transient ischemia increased astroglial reactivity using GFAP has been reported previously [5, 7, 35, 36]. Similarly, at chronic times post-insult, microglial activation detected as positive immunostaining for OX42 (an immune marker for CD11b which also stains activated neutrophils and microglial derived macrophages) [37] was increased within areas of selective necrosis at 2 and 4 weeks post insult followed by a decline in OX42 staining thereafter [5, 6, 36]. We investigated acute microglial/macrophage activation using Iba1, tomato lectin and ED1 staining and observed a consistent early (1 day) increase in Iba1 stained microglia but sparse positive ED-1 staining in 3/8 animals. Staining for activated microglia/macrophages using Iba1 and lectin staining was increased at 3 days compared to 1 day post a mild ischemic insult and ED-1 staining was present in 6/8 animals. This supports that microglial activation occurs as early as 1 day post a mild ischemic insult, consistent with previous observations of early microglial activation in peri-infarct regions i.e. regions which likely experienced mild transient ischemia; and, this is followed by the appearance of macrophages within the core and peri-infarct region at 3–5 days post-insult [37]. There is a complex and incompletely understood progression of activation of different microglial phenotypes (e.g. macrophages that are of an M1 or M2 phenotype), however, there is agreement in general that activated microglia appear to be involved in both necrotic and repair responses [21, 37–39]. Thus it is possible that in the present study, increased microglial activation observed at 3 days provides some neuroprotection and acts to help reduce damage during the second insult via expression of anti-inflammatory cytokines such as IL-10 or TGF-β and factors such as IGF-1 [21, 37, 39]. Future elucidation of the pathophysiology of the polarity changes of the various immune cells activated following ischemia should consider identifying the specific mediators within the microenvironment in response to differing severities of cerebral ischemia—including a mild transient ischemia. Presently, evidence was lacking for a potential involvement of a differential release of pro-inflammatory neurotoxic cytokines enhancing acute recurrent ischemic damage considering staining for TNF-alpha tended to be greater rather than less at 3 days.

### Systemic inflammation

Examination of peripheral blood following a short transient ischemia indicated that even a mild ischemic insult evoked alterations in inflammatory cells within the blood. Although the complex and dynamic immune responses post ischemia are still being characterized in detail and their roles in affecting ischemic outcomes remain controversial, numerous studies indicate that ischemic alterations in peripheral inflammatory cells can modify brain damage [21, 23, 24]. Following the mild transient ischemia produced currently, we found alterations in the concentrations of lymphocytes, granulocytes and platelets in the blood.

Regarding changes in lymphocytes, both our sham animals and animals 1 day post a mild ischemic insult demonstrated a decrease in peripheral lymphocytes compared to our naïve controls. This is consistent with the lymphopenia associated with immune suppression and an increased susceptibility to infection that is well recognized to occur post stroke in humans and experimental animals [40–43]. The reduction in lymphocytes with the current mild transient ischemia produced by 30 min clip occlusion was rather modest (10–15 %) and short lasting compared to the >50 % reduction in lymphocytes observed for at least 2 weeks following 60 min of transient MCAO in mice using a transient intraluminal thread occlusion [40]. Presently, lymphocyte numbers returned towards naïve control levels already by 3 days post-insult.

The white blood cell analysis also provided a count of polymorphonuclear granulocytes which consists of predominantly neutrophils along with eosinophils and basophils. The increase in granulocytes observed at 1 day post-insult is consistent with reports of increased granulocytes following stroke in humans and experimental animals [41, 43–46]. The origin of the additional granulocytes can vary with the type of injury but includes mobilization from the spleen and bone marrow and potentially increased production and decreased apoptosis [34, 37]. The mechanism mediating the increase likely includes a stress response [23, 41] which is consistent with previous reports and our observation of elevated numbers of granulocytes and decreased numbers of lymphocytes, also in animals subjected to sham surgery [40, 47, 48]. With a mild transient ischemic insult, we observed an increase in granulocytes that was temporary with a normalization by 3 days post MCAO. A similar return towards baseline was observed in mice with either sham surgery or MCAO using a transient ligature [48]. Important to note is that the stress induced neutrophilia would appear insufficient to enhance damage because a sham surgery 1 day prior to mild transient MCAO reduced ischemic damage indicating that a combination of both transient cerebral ischemia and neutrophilia would be involved in exacerbating damage.

Indeed, the interaction of granulocytes or neutrophils with cerebral ischemia is a complex operation of dynamic changes in neutrophil activation at various sites (e.g. vascular and parenchymal) underlying multiple functions (e.g. blood–brain barrier disruption, thrombus/clot formation or neurotoxicity) [34]. Both clinical and experimental studies have reported an increased accumulation of neutrophils in the brain after an ischemic stroke [34, 37, 49]; and, there is evidence for their role in enhancing brain damage but also evidence for their lack of an effect or for producing beneficial effects via neuroprotection or an involvement in brain repair e.g. [34, 37, 50–53]. This variability may reflect the array of time dependent changes in various molecular signals and their interactions on multiple cell types with potential differences according to species.

Currently, the neurovascular unit is considered a key site of neutrophil action at delayed times following transient cerebral ischemia. Following transient ischemia, Ly6G positive neutrophil infiltration into the brain parenchyma was minimal or occurred within the vicinity of vessels [54, 55]. Also following transient ischemia in the mouse, homogenized brain samples analyzed with flow cytometry demonstrated a delayed (2–3 days) increase in neutrophils not present at 1 day [53, 56]. Note that neutrophil migration appears to occur sooner and is more intense without reperfusion; it has been observed early (e.g. at 1 day) following permanent ischemia [47, 57]. Although there may be a lack of migration into brain acutely following a single transient ischemia, granulocyte recruitment, activation and infiltration [34, 37] on primed cerebral endothelium may be greatly accelerated by the subsequent flow disruption produced with a recurrent ischemic insult. A contribution from platelets is also possible as supported by their decrease at 1 day compared to 3 days post-insult possibly reflecting continued activation/interaction with cerebral ischemic vasculature. Reduced platelet counts have also been observed in stroke patients and there is evidence for their interaction with peripheral inflammatory responses and ischemic injury [58–60]. Additional research is required to clarify the importance of granulocyte and platelet interactions and the dynamic changes in injured cerebrovascular endothelium following an initial mild ischemic insult prior to a second TIA. The possibility for different peripheral immune responses occurring with a first TIA versus a recurrent stroke/TIA should also be considered [61].

## Conclusion

To conclude, the production of multiple mild ischemic insults with different recovery times between them demonstrated an important dependence on the timing between insults. The damage observed with a short recovery of 1 day following a mild transient ischemia substantially exceeded that with a subacute recovery time of 3 days. The results might help explain clinical reports of increased risk of recurrent stroke in the first day following a TIA/minor stroke or the sudden clinical deterioration observed in some cases soon after stroke [62, 63]. The mechanisms involved in the response are likely complex involving a progression of systemic and cerebral/vascular inflammatory changes after the first mild ischemic insult that affect the second insult. Irrespective, the results indicate that in the absence of clinically available therapeutic therapy to attenuate injury following a transient ischemic attack or its recurrence, patients should receive immediate care to best manage and prevent stroke recurrence e.g. with early investigation of thromboembolic sources, intensive antiplatelet therapies, lipid lowering, and/or anticoagulation for cardioembolic stroke.

## Additional files

10.1186/s12868-016-0263-x Histology assessments for Figure 1. Shown are the data for each animal for the 1d and 3d recurrent (Recur.) groups and the Sham (sh) plus 1d mild (mld) stroke group. The descriptive assessments, the H&E scores, the ED1 counts and the GFAP scores are presented.
10.1186/s12868-016-0263-x Physiological measures for each animal in the 1d and 3d recurrent mild ischemic insult groups. Shown are the doppler flow measures (% baseline), rectal temperature, mean arterial blood pressure (MABP) and blood gas values.
10.1186/s12868-016-0263-x Histological assessments for Figure 3. Shown are the data for each animal at either 1d or 3d post a single mild ischemic insult. The H&E scores, the ED1 counts and the GFAP scores are presented.
10.1186/s12868-016-0263-x Histological assessments for Figure 4. Shown are the data for each animal at either 1d or 3d post a single mild ischemic insult. Positive staining counts for TNF, Iba1 and EBA in addition to the Lectin scores and IgG gray level measures are presented.
10.1186/s12868-016-0263-x Complete blood analysis values for each of the animals in each group—the naïve control group, the control group with sham middle cerebral artery (MCA) surgery, the 1d post MCA occlusion and 3d post MCA occlusion groups. Presented are the numbers of white blood cells, lymphocytes, monocytes, granulocytes (x109/L) and the numbers of lymphocytes, monocytes and granuloctyes (% total white blood cells). Hematocrit and numbers of platelets are also presented.

## Abbreviations

EBA: endothelial barrier antigen
GFAP: glial fibrillary acidic protein
IgG: immunoglobulin G
MCAO: middle cerebral artery occlusion
TIA: transient ischemic attack
TNF: tumor necrosis factor

## References

[1] Black M, Wang W, Wang W (2015). Ischemic stroke: From next generation sequencing and GWAS to community genomics?. OMICS.

[2] Easton JD, Saver JL, Albers GW, Alberts MJ, Chaturvedi S, Feldmann E (2009). Definition and evaluation of transient ischemic attack: a scientific statement for healthcare professionals from the American Heart Association/American Stroke Association Stroke Council; Council on Cardiovascular Surgery and Anesthesia; Council on Cardiovascular Radiology and Intervention; Council on Cardiovascular Nursing; and the Interdisciplinary Council on Peripheral Vascular Disease. The American Academy of Neurology affirms the value of this statement as an educational tool for neurologists. Stroke.

[3] Kernan WN, Ovbiagele B, Black HR, Bravata DM, Chimowitz MI, Ezekowitz MD (2014). Guidelines for the prevention of stroke in patients with stroke and transient ischemic attack: a guideline for healthcare professionals from the American Heart Association/American Stroke Association. Stroke.

[4] Baron JC, Yamauchi H, Fujioka M, Endres M (2014). Selective neuronal loss in ischemic stroke and cerebrovascular disease. J Cereb Blood Flow Metab.

[5] Ejaz S, Williamson DJ, Ahmed T, Sitnikov S, Hong YT, Sawiak SJ (2013). Characterizing infarction and selective neuronal loss following temporary focal cerebral ischemia in the rat: a multi-modality imaging study. Neurobiol Dis.

[6] Ejaz S, Emmrich JV, Sawiak SJ, Williamson DJ, Baron JC (2015). Cortical selective neuronal loss, impaired behavior, and normal magnetic resonance imaging in a new rat model of true transient ischemic attacks. Stroke.

[7] Qiao M, Zhao Z, Barber PA, Foniok T, Sun S, Tuor UI (2009). Development of a model of recurrent stroke consisting of a mild transient stroke followed by a second moderate stroke in rats. J Neurosci Methods.

[8] Berkhemer OA, Fransen PS, Beumer D, van den Berg LA, Lingsma HF, Yoo AJ (2015). A randomized trial of intraarterial treatment for acute ischemic stroke. N Engl J Med.

[9] Campbell BC, Mitchell PJ, Kleinig TJ, Dewey HM, Churilov L, Yassi N (2015). Endovascular therapy for ischemic stroke with perfusion-imaging selection. N Engl J Med.

[10] Goyal M, Demchuk AM, Menon BK, Eesa M, Rempel JL, Thornton J (2015). Randomized assessment of rapid endovascular treatment of ischemic stroke. N Engl J Med.

[11] Bal S, Patel SK, Almekhlafi M, Modi J, Demchuk AM, Coutts SB (2012). High rate of magnetic resonance imaging stroke recurrence in cryptogenic transient ischemic attack and minor stroke patients. Stroke.

[12] Kappelle LJ, Van Latum JC, Van Swieten JC, Algra A, Koudstaal PJ, van Gijn J (1995). Recurrent stroke after transient ischaemic attack or minor ischaemic stroke: Does the distinction between small and large vessel disease remain true to type? Dutch TIA Trial Study Group. J Neurol Neurosurg Psychiatry.

[13] Johansson E, Cuadrado-Godia E, Hayden D, Bjellerup J, Ois A, Roquer J (2016). Recurrent stroke in symptomatic carotid stenosis awaiting revascularization: a pooled analysis. Neurology.

[14] Coutts SB, Modi J, Patel SK, Demchuk AM, Goyal M, Hill MD (2012). CT/CT angiography and MRI findings predict recurrent stroke after transient ischemic attack and minor stroke: results of the prospective CATCH study. Stroke.

[15] Clark D, Tuor UI, Thompson R, Institoris A, Kulynych A, Zhang X (2012). Protection against recurrent stroke with resveratrol: endothelial protection. PLoS ONE.

[16] Garcia JH, Liu KF, Ye ZR, Gutierrez JA (1997). Incomplete infarct and delayed neuronal death after transient middle cerebral artery occlusion in rats. Stroke.

[17] Qiao M, Meng S, Foniok T, Tuor UI (2009). Mild cerebral hypoxia-ischemia produces a sub-acute transient inflammatory response that is less selective and prolonged after a substantial insult. Int J Dev Neurosci.

[18] Ito D, Tanaka K, Suzuki S, Dembo T, Fukuuchi Y (2001). Enhanced expression of Iba1, ionized calcium-binding adapter molecule 1, after transient focal cerebral ischemia in rat brain. Stroke.

[19] Acarin L, Vela JM, Gonzalez B, Castellano B (1994). Demonstration of poly-*N*-acetyl lactosamine residues in ameboid and ramified microglial cells in rat brain by tomato lectin binding. J Histochem Cytochem.

[20] Lin B, Ginsberg MD (2000). Quantitative assessment of the normal cerebral microvasculature by endothelial barrier antigen (EBA) immunohistochemistry: application to focal cerebral ischemia. Brain Res.

[21] Iadecola C, Anrather J (2011). The immunology of stroke: from mechanisms to translation. Nat Med.

[22] Acarin L, Gonzalez B, Castro AJ, Castellano B (1999). Primary cortical glial reaction versus secondary thalamic glial response in the excitotoxically injured young brain: microglial/macrophage response and major histocompatibility complex class I and II expression. Neuroscience.

[23] Chamorro A, Meisel A, Planas AM, Urra X, van de Beek D, Veltkamp R (2012). The immunology of acute stroke. Nat Rev Neurol.

[24] Denes A, Thornton P, Rothwell NJ, Allan SM (2010). Inflammation and brain injury: acute cerebral ischaemia, peripheral and central inflammation. Brain Behav Immun.

[25] Koch S, Della-Morte D, Dave KR, Sacco RL, Perez-Pinzon MA (2014). Biomarkers for ischemic preconditioning: finding the responders. J Cereb Blood Flow Metab.

[26] Wang Y, Reis C, Applegate R, Stier G, Martin R, Zhang JH (2015). Ischemic conditioning-induced endogenous brain protection: applications pre-, per- or post-stroke. Exp Neurol.

[27] Garcia-Bonilla L, Benakis C, Moore J, Iadecola C, Anrather J (2014). Immune mechanisms in cerebral ischemic tolerance. Front Neurosci.

[28] Esposito E, Hayakawa K, Maki T, Arai K, Lo EH (2015). Effects of postconditioning on neurogenesis and angiogenesis during the recovery phase after focal cerebral ischemia. Stroke.

[29] Hanyu S, Ito U, Hakamata Y, Yoshida M (1995). Transition from ischemic neuronal necrosis to infarction in repeated ischemia. Brain Res.

[30] Hanyu S, Ito U, Hakamata Y, Nakano I (1997). Topographical analysis of cortical neuronal loss associated with disseminated selective neuronal necrosis and infarction after repeated ischemia. Brain Res.

[31] Coutts SB, Modi J, Patel SK, Aram H, Demchuk AM, Goyal M (2012). What causes disability after transient ischemic attack and minor stroke? Results from the CT and MRI in the Triage of TIA and minor Cerebrovascular Events to Identify High Risk Patients (CATCH) Study. Stroke.

[32] Zhao L, Nowak TS (2006). CBF changes associated with focal ischemic preconditioning in the spontaneously hypertensive rat. J Cereb Blood Flow Metab.

[33] Hoyte LC, Papadakis M, Barber PA, Buchan AM (2006). Improved regional cerebral blood flow is important for the protection seen in a mouse model of late phase ischemic preconditioning. Brain Res.

[34] Jickling GC, Liu D, Ander BP, Stamova B, Zhan X, Sharp FR (2015). Targeting neutrophils in ischemic stroke: translational insights from experimental studies. J Cereb Blood Flow Metab.

[35] Arsava EM, Gurer G, Gursoy-Ozdemir Y, Karatas H, Dalkara T (2009). A new model of transient focal cerebral ischemia for inducing selective neuronal necrosis. Brain Res Bull.

[36] Hughes JL, Beech JS, Jones PS, Wang D, Menon DK, Baron JC (2010). Mapping selective neuronal loss and microglial activation in the salvaged neocortical penumbra in the rat. Neuroimage.

[37] Benakis C, Garcia-Bonilla L, Iadecola C, Anrather J (2014). The role of microglia and myeloid immune cells in acute cerebral ischemia. Front Cell Neurosci.

[38] Perego C, Fumagalli S, De Simoni MG (2011). Temporal pattern of expression and colocalization of microglia/macrophage phenotype markers following brain ischemic injury in mice. J Neuroinflammation.

[39] Patel AR, Ritzel R, McCullough LD, Liu F (2013). Microglia and ischemic stroke: a double-edged sword. Int J Physiol Pathophysiol Pharmacol.

[40] Prass K, Meisel C, Hoflich C, Braun J, Halle E, Wolf T (2003). Stroke-induced immunodeficiency promotes spontaneous bacterial infections and is mediated by sympathetic activation reversal by poststroke T helper cell type 1-like immunostimulation. J Exp Med.

[41] Dirnagl U, Klehmet J, Braun JS, Harms H, Meisel C, Ziemssen T (2007). Stroke-induced immunodepression: experimental evidence and clinical relevance. Stroke.

[42] Urra X, Cervera A, Villamor N, Planas AM, Chamorro A (2009). Harms and benefits of lymphocyte subpopulations in patients with acute stroke. Neuroscience.

[43] Vogelgesang A, Grunwald U, Langner S, Jack R, Broker BM, Kessler C (2008). Analysis of lymphocyte subsets in patients with stroke and their influence on infection after stroke. Stroke.

[44] Buck BH, Liebeskind DS, Saver JL, Bang OY, Yun SW, Starkman S (2008). Early neutrophilia is associated with volume of ischemic tissue in acute stroke. Stroke.

[45] Ross AM, Hurn P, Perrin N, Wood L, Carlini W, Potempa K (2007). Evidence of the peripheral inflammatory response in patients with transient ischemic attack. J Stroke Cerebrovasc Dis.

[46] Ito U, Hakamata Y, Kawakami E, Oyanagi K (2011). Temporary [corrected] cerebral ischemia results in swollen astrocytic end-feet that compress microvessels and lead to delayed [corrected] focal cortical infarction. J Cereb Blood Flow Metab.

[47] Moller K, Boltze J, Posel C, Seeger J, Stahl T, Wagner DC (2014). Sterile inflammation after permanent distal MCA occlusion in hypertensive rats. J Cereb Blood Flow Metab.

[48] Denes A, Pradillo JM, Drake C, Buggey H, Rothwell NJ, Allan SM (2014). Surgical manipulation compromises leukocyte mobilization responses and inflammation after experimental cerebral ischemia in mice. Front Neurosci.

[49] Price CJ, Menon DK, Peters AM, Ballinger JR, Barber RW, Balan KK (2004). Cerebral neutrophil recruitment, histology, and outcome in acute ischemic stroke: an imaging-based study. Stroke.

[50] Krams M, Lees KR, Hacke W, Grieve AP, Orgogozo JM, Ford GA (2003). Acute Stroke Therapy by Inhibition of Neutrophils (ASTIN): an adaptive dose-response study of UK-279,276 in acute ischemic stroke. Stroke.

[51] Allen C, Thornton P, Denes A, McColl BW, Pierozynski A, Monestier M (2012). Neutrophil cerebrovascular transmigration triggers rapid neurotoxicity through release of proteases associated with decondensed DNA. J Immunol.

[52] Posel C, Scheibe J, Kranz A, Bothe V, Quente E, Frohlich W (2014). Bone marrow cell transplantation time-dependently abolishes efficacy of granulocyte colony-stimulating factor after stroke in hypertensive rats. Stroke.

[53] Garcia-Bonilla L, Moore JM, Racchumi G, Zhou P, Butler JM, Iadecola C (2014). Inducible nitric oxide synthase in neutrophils and endothelium contributes to ischemic brain injury in mice. J Immunol.

[54] Enzmann G, Mysiorek C, Gorina R, Cheng YJ, Ghavampour S, Hannocks MJ (2013). The neurovascular unit as a selective barrier to polymorphonuclear granulocyte (PMN) infiltration into the brain after ischemic injury. Acta Neuropathol.

[55] Ullrich N, Strecker JK, Minnerup J, Schilling M (2014). The temporo-spatial localization of polymorphonuclear cells related to the neurovascular unit after transient focal cerebral ischemia. Brain Res.

[56] Gelderblom M, Leypoldt F, Steinbach K, Behrens D, Choe CU, Siler DA (2009). Temporal and spatial dynamics of cerebral immune cell accumulation in stroke. Stroke.

[57] Chu HX, Kim HA, Lee S, Moore JP, Chan CT, Vinh A (2014). Immune cell infiltration in malignant middle cerebral artery infarction: comparison with transient cerebral ischemia. J Cereb Blood Flow Metab.

[58] Tohgi H, Suzuki H, Tamura K, Kimura B (1991). Platelet volume, aggregation, and adenosine triphosphate release in cerebral thrombosis. Stroke.

[59] D’Erasmo E, Aliberti G, Celi FS, Romagnoli E, Vecci E, Mazzuoli GF (1990). Platelet count, mean platelet volume and their relation to prognosis in cerebral infarction. J Intern Med.

[60] Denes A, Pradillo JM, Drake C, Sharp A, Warn P, Murray KN (2014). *Streptococcus pneumoniae* worsens cerebral ischemia via interleukin 1 and platelet glycoprotein Ibalpha. Ann Neurol.

[61] Ross AM, Lee CS (2015). Description and identification of the peripheral immune response trajectories over time in first-time and recurrent stroke/transient ischemic attack. J Neurosci Nurs.

[62] Awadh M, MacDougall N, Santosh C, Teasdale E, Baird T, Muir KW (2010). Early recurrent ischemic stroke complicating intravenous thrombolysis for stroke: incidence and association with atrial fibrillation. Stroke.

[63] Kennedy J, Hill MD, Ryckborst KJ, Eliasziw M, Demchuk AM, Buchan AM (2007). Fast assessment of stroke and transient ischaemic attack to prevent early recurrence (FASTER): a randomised controlled pilot trial. Lancet Neurol.

